# Supplementary material for: A Small Molecule Stabilizes the Disordered Native State of the Alzheimer’s Aβ Peptide
Source: ACS Chem Neurosci. 2022 Jun 1;13(12):1738–45. doi: 10.1021/acschemneuro.2c00116 (PMC9204762; doi:10.1021/acschemneuro.2c00116)
Supplement: Supplementary file 1 — cn2c00116_si_001.pdf [file cn2c00116_si_001.pdf]

Supporting Information: A small molecule stabilises the disordered native state of the Alzheimer's Aβ peptide

Thomas Löhr,[†] Kai Kohlhoff,[‡] Gabriella T. Heller,[¶,†] Carlo Camilloni,[§] and Michele Vendruscolo[*,†]

†*Department of Chemistry, University of Cambridge, Cambridge, UK*

‡*Google Research, Mountain View, CA, USA*

¶*Department of Structural and Molecular Biology, University College London, London, UK*

§*Dipartimento di Bioscienze, Università degli Studi di Milano, Milano, Italy*

E-mail: mv245@cam.ac.uk

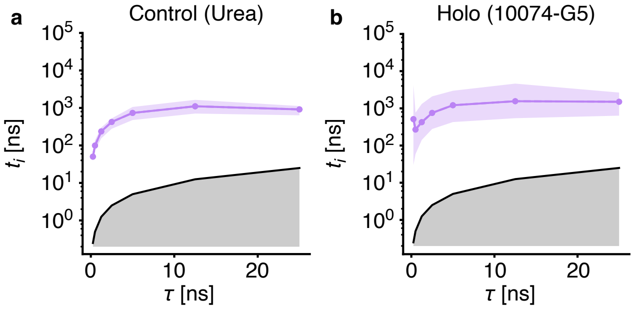

Figure S1: Relaxation timescale as a function of model lag time for **a** control (urea) and **b** holo (10074-G5) ensembles. Gray shaded areas indicate timescales the Koopman model can no longer resolve. Coloured shaded areas indicate 95th percentiles of the sample mean over all 20 models.

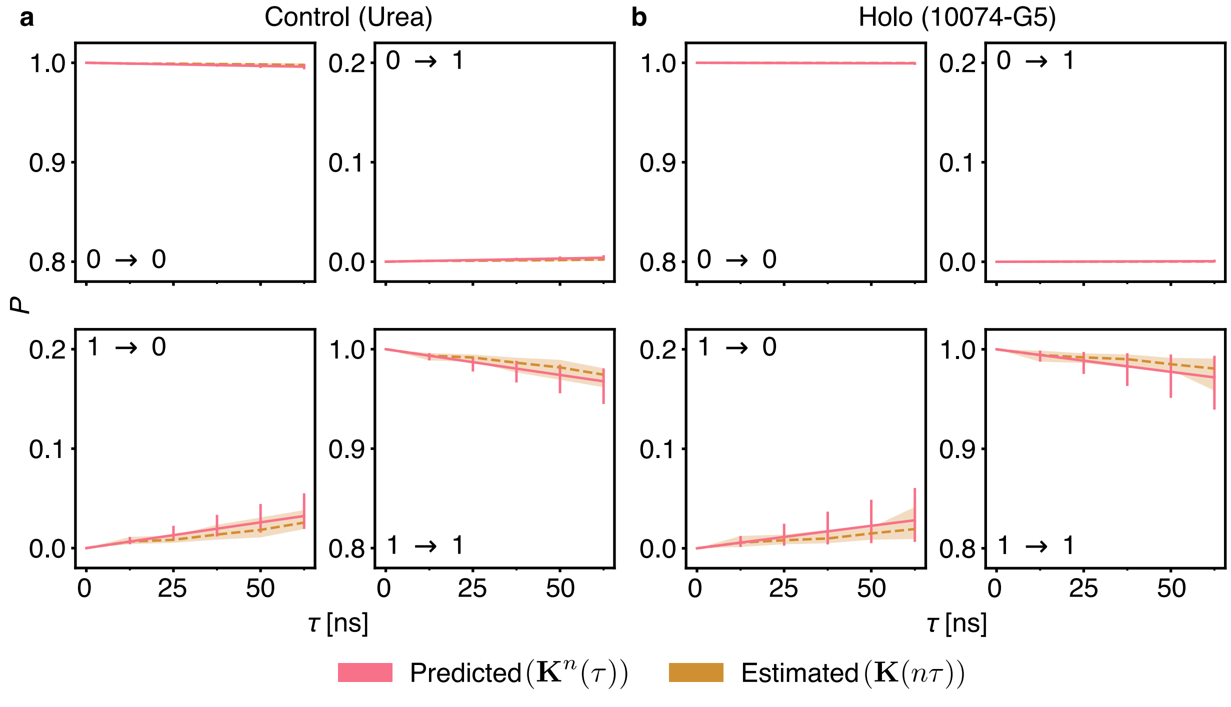

Figure S2: Chapman-Kolmogorov test for **a** control (urea) and **b** holo (10074-G5) ensembles. Each panel indicates the transition probability for one matrix entry for successive applications (predicted, red) and estimations (estimated, orange) of the Koopman matrix. Shaded areas and error bars indicate 95th percentiles of the mean over all 20 models.

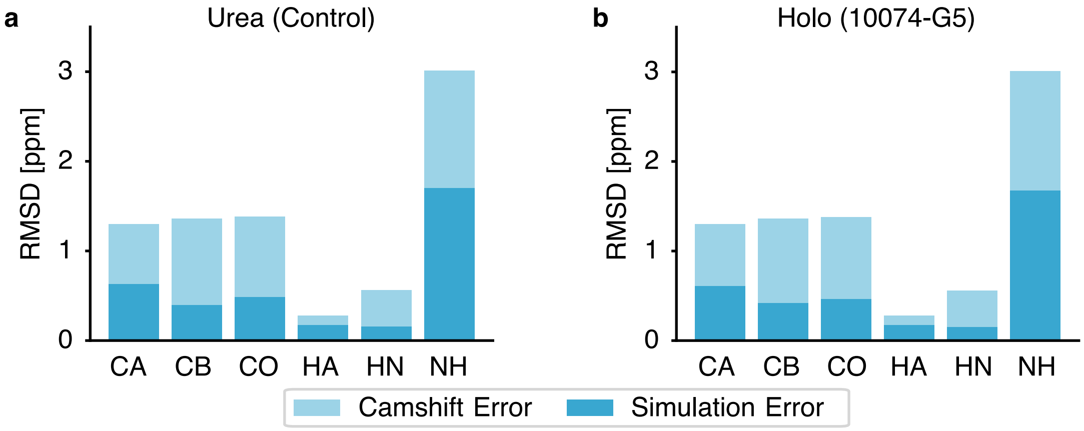

Figure S3: Root-mean-square deviations between experimentally determined NMR chemical shifts? and those back-calculated using CamShift? for **a** control (urea) and **b** holo (10074-G5) ensembles. Light shaded areas indicate the intrinsic error of the CamShift predictor.

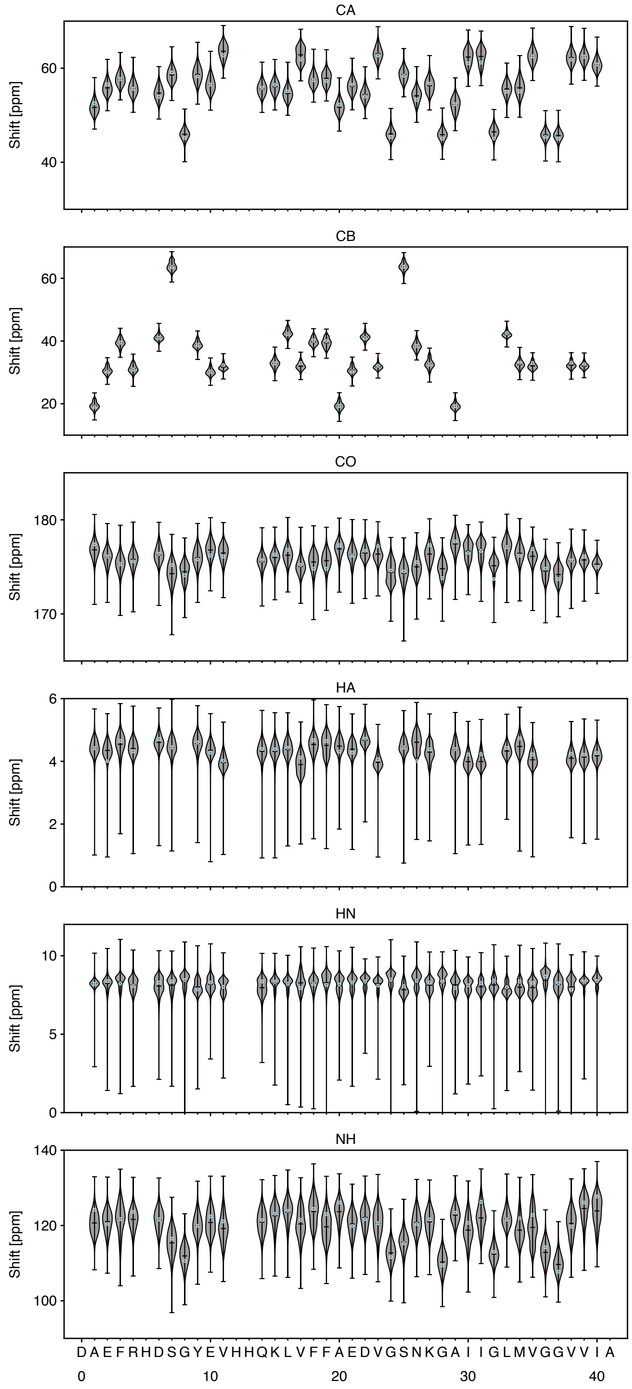

Figure S4: Distributions of back-calculated chemical shifts for each residue and atom-type for the previously determined apo (no small molecule) ensemble[?] using CamShift.[?] The horizontal bar indicates the distribution mean, the dot corresponds to the experimental value,[?] and violin whiskers indicate 95th percentiles.

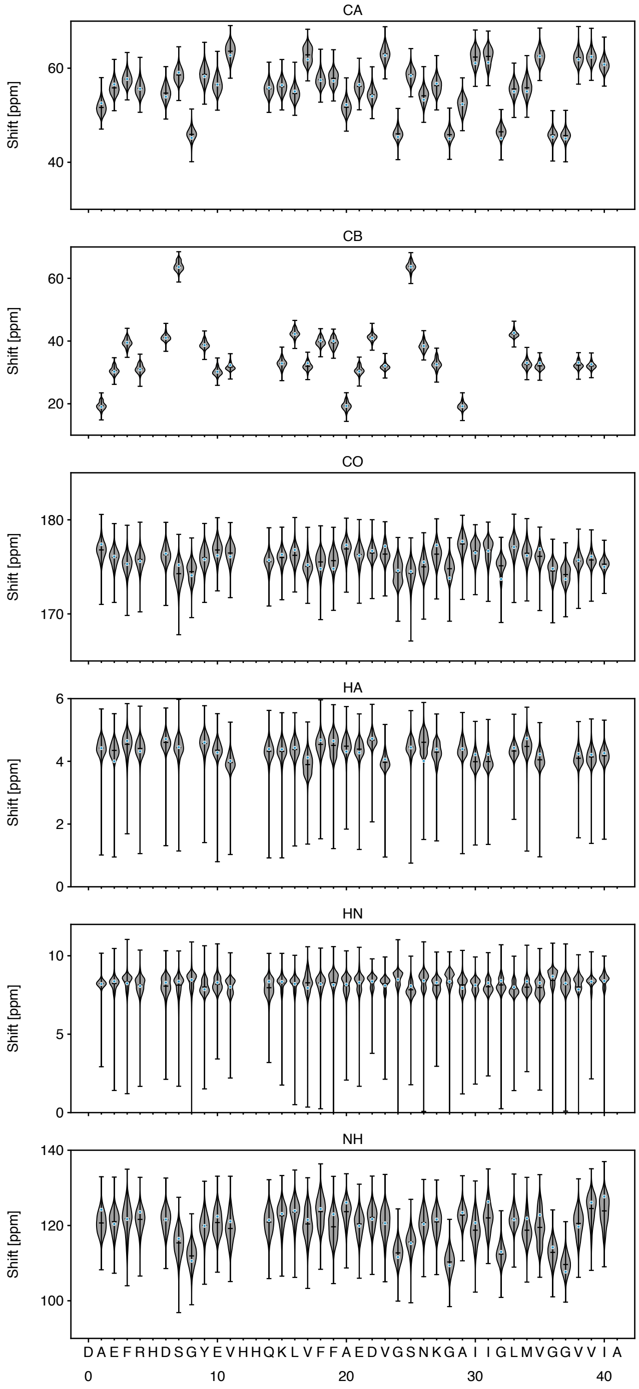

Figure S5: Distributions of back-calculated chemical shifts for each residue and atom-type for the control (urea) ensemble using CamShift.[?] The horizontal bar indicates the distribution mean, the dot corresponds to the experimental value,[?] and violin whiskers indicate 95th percentiles.

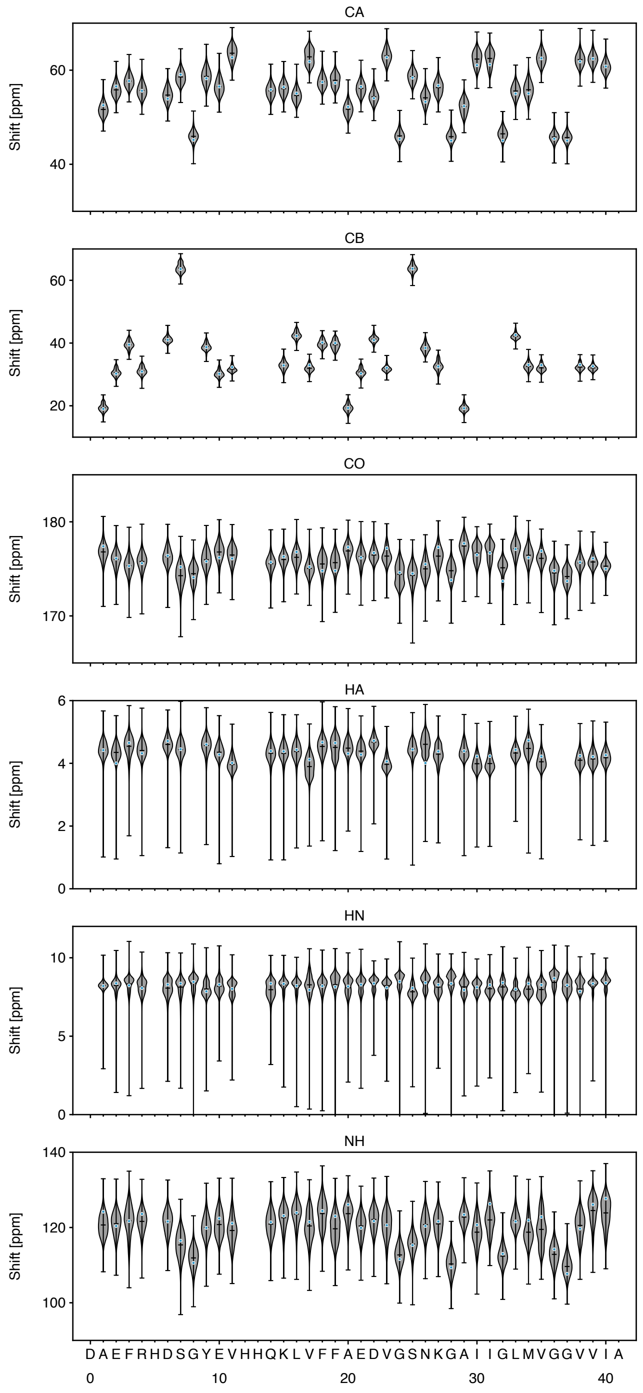

Figure S6: Distributions of back-calculated chemical shifts for each residue and atom-type for the holo (10074-G5) ensemble using CamShift.[?] The horizontal bar indicates the distribution mean, the dot corresponds to the experimental value,[?] and violin whiskers indicate 95th percentiles.

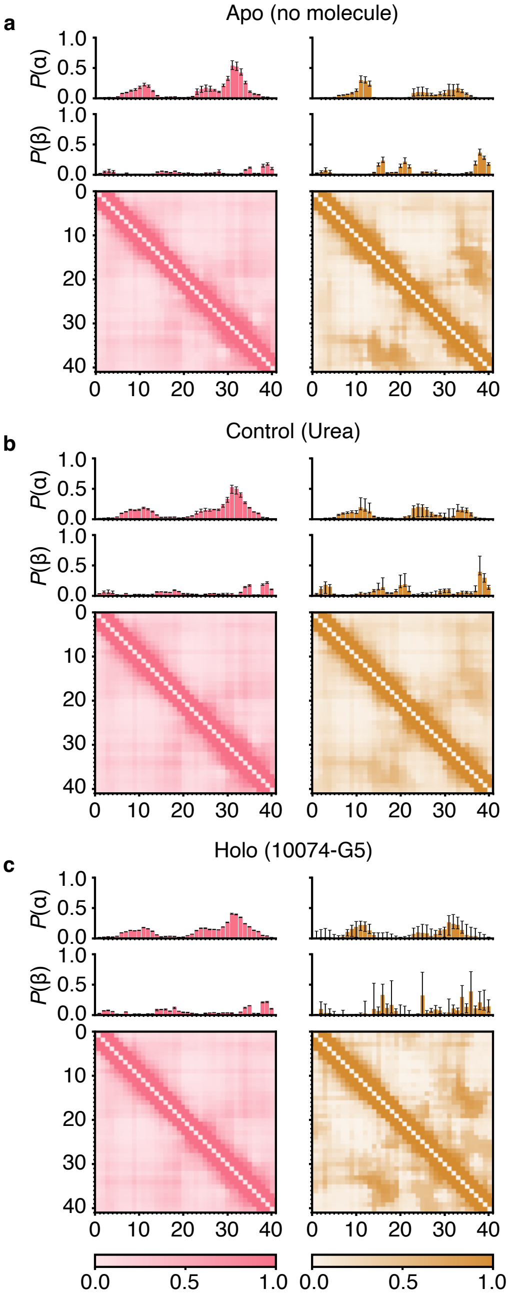

Figure S7: Structural properties of the **a** apo (no small molecule, previously determined[?]), **b** control (urea) and **c** holo (10074-G5) ensembles. Top panels indicate α-helical and β-sheet contents over all residues as calculated using DSSP.[?] Bottom panels show heavy-atom contact probability maps with a cut-off of 0.8 nm. Error bars indicate 95th percentiles of the bootstrap sample of the mean over all 20 models.

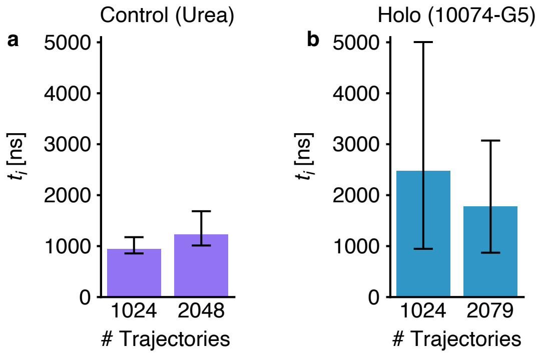

Figure S8: Dependence of the relaxation timescales on the number of trajectories used to build the model. Error bars indicate 95th percentiles of the bootstrap sample of the mean over the first 5 models.

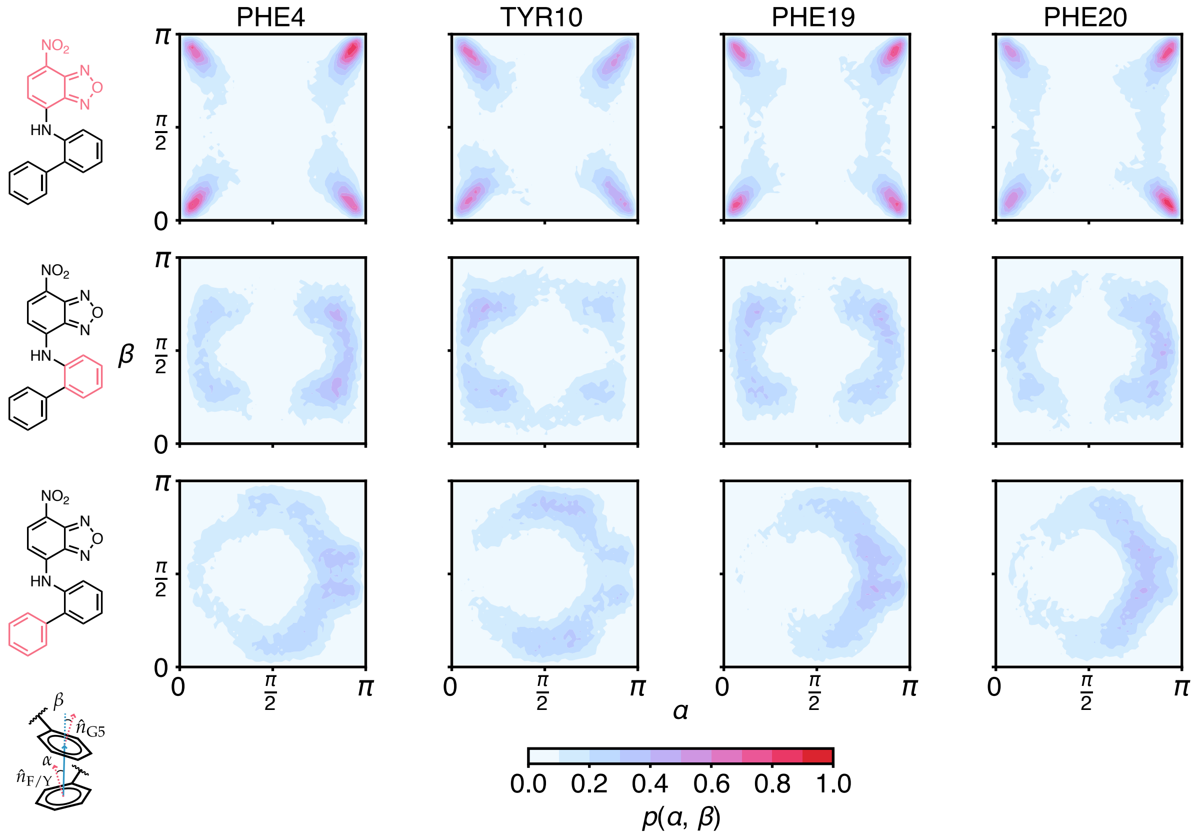

Figure S9: Anisotropy of π-π molecule — aromatic side chain stacking interactions for the holo (10074-G5) ensemble. α is the stacking angle between the inter-aromatic distance vector and the aromatic side chain normal vector, while β is the angle inter-aromatic distance vector and the normal vector of the small molecule aromatic system.? Distributions show the density under the condition that the distance between both groups is below 0.6 nm.
